# Prokaryotic Nucleotide Composition Is Shaped by Both Phylogeny and the Environment

**DOI:** 10.1093/gbe/evv063

**Published:** 2015-04-09

**Authors:** Erin R. Reichenberger, Gail Rosen, Uri Hershberg, Ruth Hershberg

**Affiliations:** ^1^Department of Biomedical Engineering, Science & Health Systems, Drexel University; ^2^Department of Computer and Electrical Engineering, Drexel University; ^3^Department of Microbiology and Immunology, Drexel University College of Medicine; ^4^Rachel and Menachem Mendelovitch Evolutionary Processes of Mutation and Natural Selection Research Laboratory, Department of Genetics and Developmental Biology, The Ruth and Bruce Rappaport Faculty of Medicine, Technion-Israel Institute of Technology, Haifa, Israel

**Keywords:** GC-content, metagenomics, evolutionary forces, mutation, natural selection, genomic variation

## Abstract

The causes of the great variation in nucleotide composition of prokaryotic genomes have long been disputed. Here, we use extensive metagenomic and whole-genome data to demonstrate that both phylogeny and the environment shape prokaryotic nucleotide content. We show that across environments, various phyla are characterized by different mean guanine and cytosine (GC) values as well as by the extent of variation on that mean value. At the same time, we show that GC-content varies greatly as a function of environment, in a manner that cannot be entirely explained by disparities in phylogenetic composition. We find environmentally driven differences in nucleotide content not only between highly diverged environments (e.g., soil, vs. aquatic vs. human gut) but also within a single type of environment. More specifically, we demonstrate that some human guts are associated with a microbiome that is consistently more GC-rich across phyla, whereas others are associated with a more AT-rich microbiome. These differences appear to be driven both by variations in phylogenetic composition and by environmental differences—which are independent of these phylogenetic composition differences. Combined, our results demonstrate that both phylogeny and the environment significantly affect nucleotide composition and that the environmental differences affecting nucleotide composition are far subtler than previously appreciated.

## Introduction

What determines nucleotide composition is an area of intense study in both prokaryotes and eukaryotes (e.g., Sueoka 1962; Bernardi and Bernardi 1986; Ikehara et al. 1996; Fullerton et al. 2001; Naya et al. 2002; Bentley and Parkhill 2004; Bernaola-Galvan et al. 2004; Foerstner et al. 2005; Matallana-Surget et al. 2008; Barkovsky and Khrustalev 2009; Touchon et al. 2009; Hershberg and Petrov 2010; Hildebrand et al. 2010; Wu et al. 2012, 2014; Chen et al. 2013). The degree of variation in nucleotide content is particularly expansive in prokaryotes where it ranges from approximately 15% to approximately 75% guanine and cytosine (GC) (Sueoka 1962; Bentley and Parkhill 2004; Nakabachi et al. 2006). Yet, currently there is no general agreement on what causes the extensive variation in nucleotide content among prokaryotes. Explanations fall into two broad categories: Neutral processes and natural selection. Neutral explanations suggest that variation in genomic nucleotide composition arises due to neutral processes, such as mutation and/or biased gene conversion (BGC). In contrast, it has also been suggested that natural selection exerted by environmental factors may be responsible for generating this variation. As variation in nucleotide content is a genome-wide trait that affects the genome as a whole, it is highly interesting to understand what drives such variation, and whether it is driven by neutral processes or by natural selection.

Mutation may drive variation in nucleotide content, if different bacterial lineages vary in their mutational biases. Under such a model, GC-biased mutational patterns will generate GC-rich organisms, whereas AT-biased mutational patterns will generate AT-rich organisms. Fitting with this model, studies have shown a possible correlation between the DNA polymerase subunit content of bacterial genomes and their GC-content (Wu et al. 2014). However, the model by which mutational biases drive variation in nucleotide content was recently driven out of favor by the discovery that mutation is universally AT-biased—even in prokaryotes with highly GC-rich genomes (Hershberg and Petrov 2010; Hildebrand et al. 2010). A second neutral process, suggested to affect GC-content, is BGC (Duret and Galtier 2009). It has been demonstrated that gene conversion is GC-biased in many eukaryotes, and in such cases, the probability that a GC allele is passed on to the next generation through gene conversion is higher than that of an AT allele. As a result of such BGC, in these eukaryotes, regions with lower recombination rates tend to be more AT-rich, whereas regions undergoing more recombination will tend to be more GC-rich (Fullerton et al. 2001). A relationship between levels of recombination and GC-content was recently demonstrated for many bacteria, suggesting that BGC, or a mechanism similar to BGC, may affect nucleotide content in bacteria in a similar manner (Touchon et al. 2009; Lassalle et al. 2015). Under this model, prokaryotes will tend to be more GC-rich if they have higher rates of recombination, higher effective population sizes, and/or a process of gene conversion that is more biased toward GC.

One can easily imagine that neutral processes affecting nucleotide composition may vary between different bacterial lineages. It may appear less likely that these neutral processes would vary between environments in a systematic manner. Therefore, it may be a reasonable prediction that neutral processes contribute to variation in nucleotide content between phyla, but not between environments. An observation of environmentally driven variation in nucleotide content, which cannot be explained solely by differences in phylogenetic composition, will therefore indicate a role for natural selection in driving this variation. In other words, it would indicate that certain environmental factors select for certain nucleotide compositions.

Several studies have investigated whether certain environmental factors provoke distinct GC-levels. Correlations between GC-content and aerobiosis, environmental temperature, radiation levels, or the presence of exogenous entities have been noted (Moran 2002; Naya et al. 2002; Basak and Ghosh 2005; Navarre et al. 2006; Matallana-Surget et al. 2008; Mendez et al. 2010; Raghavan et al. 2012; Wu et al. 2012). Evidence for the influence of specific environmental factors on GC-content remains inconclusive (Galtier and Lobry 1997; Bentley and Parkhill 2004; Basak and Ghosh 2005; Wang et al. 2006; Zhao et al. 2007; Hildebrand et al. 2010; Chen et al. 2013; Agashe and Shankar 2014).

In order to determine whether the environment is a contributing factor to DNA composition, it is highly useful to compare the GC-content of microbes extracted from a broad array of environments. The recent availability of shotgun sequenced metagenomic data allows for such an examination. A previous study from Foerstner et al. (2005) utilized four metagenomic data sets extracted from an equal number of radically distinct environments: Ocean surface water, farm soil, mine drainage biofilm, and deep sea whale carcass. Using these data sets, Foerstner et al. demonstrated that the bacterial community in one environment carried a GC signature that was distinct from bacterial communities in the other environments. As these signatures could not be entirely explained by environmental differences in phylogenetic composition, they concluded that the environment was responsible for the observed inter-environmental variation in nucleotide composition. However, a major drawback to their research was the limited number of available metagenomic samples. This allowed Foerstner et al. to compare only between one or two samples extracted from four very different environments. Thus, both due to the paucity of their examples and because they could not compare between samples extracted from more similar environments, the generality of their results was unclear. Specifically, they missed the complexity of phylogenetic and environmental impacts we describe here.

Employing numerous shotgun-sequenced data sets as well as data from all currently available fully sequenced genomes, we show that both phylogeny and environment influence prokaryotic nucleotide composition. First, we show that, across environments, different phyla have distinct nucleotide compositions. We then show that GC-levels vary by environment in a manner that cannot be explained solely by differences in phylogenetic composition. Furthermore, we observed that environmentally influenced variation in GC-composition is found not only between drastically different environments, such as soil and water, but also within samples of a single type of environment (e.g., in our analysis of multiple human gut samples). Thus, the environmental factors influencing nucleotide content seem to be far subtler than previously appreciated.

## Materials and Methods

### Data Sources

Shotgun-sequenced fasta files from numerous environments were obtained from MG-Rast (Meyer et al. 2008). Details of each project’s methodology, metadata, and geographic location can be found here: http://simlab.biomed.drexel.edu/maps/GC_map.php (Edwards et al. 2006; Wegley et al. 2007; Desnues et al. 2008; Dinsdale, Edwards, et al. 2008; Dinsdale, Pantos, et al. 2008; Kunin et al. 2008; Mou et al. 2008; Angly et al. 2009; Rodriguez-Brito et al. 2010; Swan et al. 2010; Belda-Ferre et al. 2012; Pride et al. 2012; Yatsunenko et al. 2012). The files were downloaded at the screened level in the analysis process, which should have excluded ambiguous reads, short sequence reads, low quality scores, and redundant sequences. Additionally, reads shorter than 100 bp were removed from consideration. The remaining reads were then taxonomically classified (from the level of phylum to that of genus) using the PhymmBL software (Brady and Salzberg 2009, 2011). Reads classified at the phylum level with a confidence score of 80% or higher were then analyzed for their GC-content. In totum, 33 unique prokaryotic phyla were identified and their relative abundance was calculated for each data set. Using the relative abundance, attention was focused on the ten phyla that consistently appeared to be most prevalent across all data sets. These phyla included two archaea, Euryarchaeota and Crenarchaeota along with eight bacterial phyla (Actinobacteria, Bacteroidetes, Chlamydiae, Deinococcus-Thermus, Firmicutes, Proteobacteria, Spirochaetes, and Tenericutes).

Full genome sequences and their taxonomic classifications were downloaded in October 2014 from the NCBI (National Center for Biotechnology Information) microbial database (Pruitt et al. 2007).

In order to examine the levels of sequence variation of orthologous protein pairs within each phylum, we used the POGO database (Lan et al. 2014).

### Assessing Genus-Level Similarity between Environments

In order to assess how similar two environments were in the identities of the genera they contained, sequences within each environment were classified at the genera level. We then created for each environment a list of the genera that were present within that environment. The lists from different environments were compared by calculating the Jaccard similarity coefficient, which is defined as the union of the two sets (how many genera are contained within the two environments together) divided by the intersection of the two sets (how many genera are shared by the two environments) (eq. 1) (Levandowsky and Winter 1971).

(1) $$\begin{array}{c} \text{Jaccard}({\mathrm{Environment}}_{A},{\mathrm{Environment}}_{B})\\ =\frac{\left|{\mathrm{Environment}}_{A}\cap {\mathrm{Environment}}_{B}\right|}{\left|{\mathrm{Environment}}_{A}\cup {\mathrm{Environment}}_{B}\right|}. \end{array}$$

### Annotation of Protein-Coding Genes and Extraction of 4-Fold Degenerate Third-Codon Positions

Sequences with a confidence score (PhymmBL) ≥ 80% were run through FragGeneScan for gene detection (Rho et al. 2010). Each successfully annotated sequence was examined for the location of those amino acids with 4-fold redundancies (Alanine, Arginine, Glycine, Leucine, Proline, Serine, Threonine, and Valine). Third-codon positions of these codons were then extracted for GC-content calculations.

The similarity analysis, Wilcoxon Rank Sum Test (Mann–Whitney–Wilcoxon), and hypergeometric probability were done in Python, all other statistical analyses were performed with the R-statistical package (van Rossum 1995; R Core Team 2013).

## Results

### Mean GC-Content and Degree of Variation in GC-Content Vary Greatly between Prokaryotic Phyla

**Fig. 1.—:**
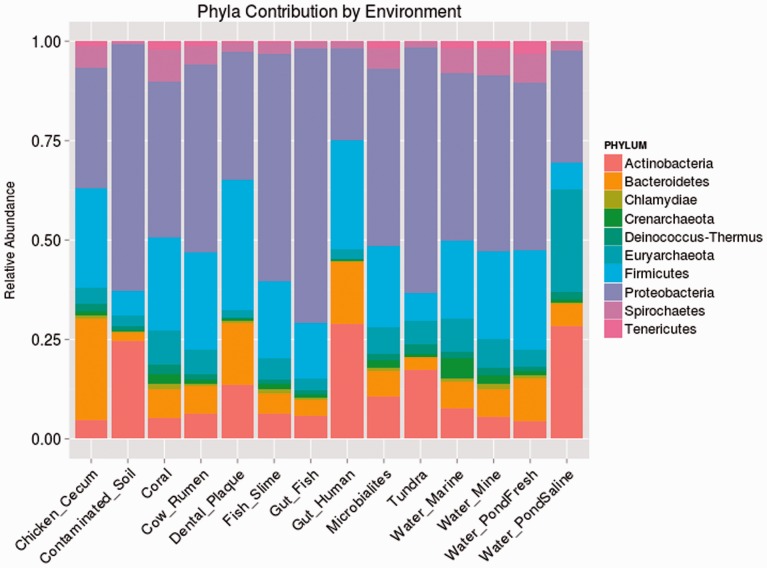
Relative abundance of each phylum within the various sampled environments.

Taxonomic analysis and GC-content assessment were performed on over 31 million sequences from 183 shotgun-sequenced metagenomic data sets, which were taken from 14 types of environments (table 1). The number of data sets along with the raw and relative abundance of sequences for the ten phyla which were the most abundant across all environments (see Materials and Methods) can be seen in table 1 and figure 1. Supplementary figure S1, Supplementary Material online, contains the plots for the distribution of GC% by taxon in each environment.

**Table 1:** Number of Data Sets and Sequences by Environment

Environment	Number of Data Sets	Number of Sequences^a^
Chicken Cecum	2	384,676
Contaminated Soil	3	3,654,826
Coral	7	427,591
Cow Rumen	3	490,767
Dental Plaque	8	1,725,397
Fish Slime	2	80,878
Fish Gut	2	57,122
Human Gut	111	16,047,825
Microbialites	13	515,358
Tundra	1	5,894,070
Water Marine	13	810,607
Water Mine	2	359,534
Water PondFresh	4	325,037
Water PondSaline	12	665,214
Total	183	31,438,902

^a^Number of classified sequences.

Based on mean GC-content, phyla could be classified into one of three categories: GC-rich (Actinobacteria: 62.1%, Deinococcus-Thermus: 64.4%), GC-intermediate (Crenarchaeota: 49.7%, Euryarchaeota: 53.7%, Proteobacteria: 56.4%), and GC-poor (Bacteroidetes: 46.0%, Chlamydiae: 40.3%, Firmicutes: 43.1%, Spirochaetes: 40.6%, Tenericutes: 32.2%). The GC composition of the phyla within the metagenomic data sets coincided with those of the referential fully sequenced genomes of the same phyla (supplementary fig. S3, Supplementary Material online). A phylogenetic tree based on species from the selected phyla and represents the relationship from one phylum to another can be seen in supplementary figure S4, Supplementary Material online (Letunic and Bork 2011; Sharpton 2014).

Levels of variation in GC-content around the calculated mean also differed greatly among the phyla. Certain phyla showed great variability in their range of GC-levels (e.g., Spirochaetes: ±11.8) whereas other phyla had moderate consistency (e.g., Tenericutes: ±8.9). Strikingly, Chlamydiae and Deinococcus-Thermus seemed highly impervious to their surroundings and maintained a very restricted GC-composition with a standard deviation of approximately ±5.5 and ±5.9, respectively. Whether a certain phylum had a broad or narrow range of GC-compositions, it tended to be consistent across environments (supplementary fig. S1, Supplementary Material online). In other words, phyla that had a broad range of GC-compositions in one environment tended to have a similarly broad range in all remaining environments, whereas phyla that had a narrow range tended to consistently present a narrow range in the other environments.

The homogeneous GC-levels seen, respectively, within Chlamydiae and Deinococcus-Thermus could be the result of low sequence divergence between members of these phyla. To examine this possibility, we calculated the average amino acid identity (AAI) levels of orthologous protein-coding genes belonging to fully sequenced members of each phyla (supplementary fig. S5, Supplementary Material online). The plots show that for Chlamydiae and Deinococcus-Thermus, there are numerous genome pairs that are highly diverged. Levels of divergence do not seem to be lower for these phyla than for other phyla that have much higher GC-ranges. These results suggest that low levels of nucleotide content variation within these lineages are not due to low levels of sequence variation within the same lineages.

### GC-Content Varies across Environments in a Manner that Cannot Be Explained by Differences in Phylogeny

**Fig. 2.—:**
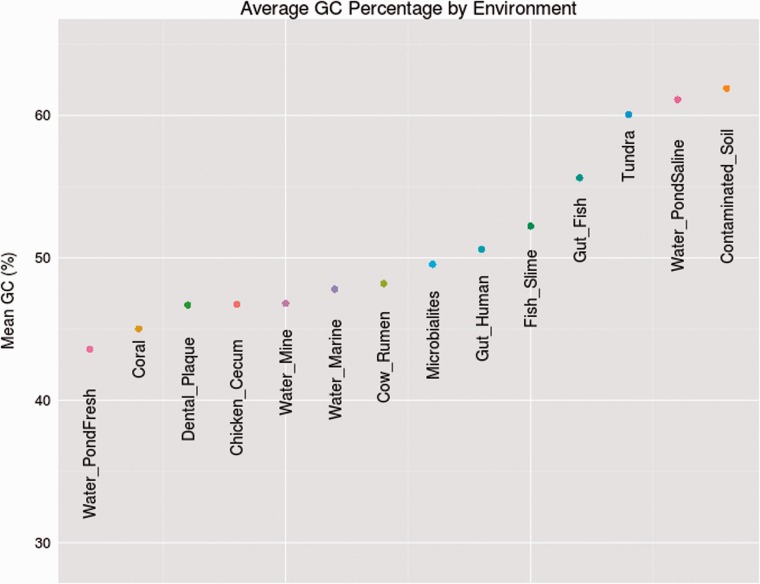
Average GC-composition by environment. The GC-composition was averaged across the ten phyla found to be most abundant across all sampled biomes in an environmental category.

As can be seen in figure 2 and its corresponding box plot in supplementary figure S2, Supplementary Material online, GC-levels vary by environment. To rule out the possibility that variation in GC-composition between environments could be explained entirely by differences in phylogenetic composition, we examined whether the nucleotide content of different phyla correlated across environments. Such correlations would indicate that whatever force influenced the nucleotide content in one phylum had a similar effect on the nucleotide content of the remaining phyla. This would demonstrate that variation in GC-content between environmental categories was not the sole result of distinct phylogenetic compositions in different metagenomic samples. Rather, it would indicate that phyla were affected by their environment in a similar, correlated manner.

To determine whether there was a correlative relationship between the GC-content of each phyla, the Spearman correlation coefficients (with significance) were calculated. We first looked at the correlations (table 2*A*) for the binned mean GC-values for each phylum (*n* = 10) in every data set (*n* = 183). The GC-contents of all phyla were significantly correlated (*P* ≪ 0.05) to those of other phyla, much more often than the 5% expected by chance. The GC-content of Deinococcus-Thermus was significantly correlated to that of other phyla in 56% of all cases. The GC-contents of the remaining phyla were significantly correlated to those of the remaining phyla 78–100% of the time (table 2*A*).

**Table 2:** Spearman Correlation Coefficients for (*A*) All Environments, (*B*) Human Gut Environment, and (*C*) All Environments minus the Human Gut

	Act	Bact	Chl	Cren	DT	Eury	Firm	Pro	Spiro	Ten
(*A*)										
Act	1	0.169*	−0.084	0.216*	0.661*	0.56*	0.032	0.56*	−0.203*	−0.213*
Bact	0.169*	1	0.544*	0.458*	0.161*	0.596*	0.758*	0.607*	0.661*	0.26*
Chl	−0.084	0.544*	1	0.429*	−0.108	0.325*	0.614*	0.235*	0.688*	0.457*
Cren	0.216*	0.458*	0.429*	1	0.121	0.683*	0.265*	0.296*	0.318*	0.259*
DT	0.661*	0.161*	−0.108	0.121	1	0.304*	0.104	0.394*	−0.13	−0.227*
Eury	0.56*	0.596*	0.325*	0.683*	0.304*	1	0.296*	0.621*	0.249*	0.137
Firm	0.032	0.758*	0.614*	0.265*	0.104	0.296*	1	0.477*	0.774*	0.366*
Pro	0.56*	0.607*	0.235*	0.296*	0.394*	0.621*	0.477*	1	0.245*	−0.067
Spiro	−0.203*	0.661*	0.688*	0.318*	−0.13	0.249*	0.774*	0.245*	1	0.601*
Ten	−0.213*	0.26*	0.457*	0.259*	−0.227*	0.137	0.366*	−0.067	0.601*	1
(*B*)										
Act	1	0.016	0.085	0.048	0.444*	0.248*	0.056	0.394*	0.075	0.063
Bact	0.016	1	0.575*	0.759*	0.022	0.716*	0.627*	0.51*	0.804*	0.347*
Chl	0.085	0.575*	1	0.637*	−0.008	0.566*	0.555*	0.282*	0.725*	0.349*
Cren	0.048	0.759*	0.637*	1	−0.048	0.717*	0.559*	0.423*	0.779*	0.441*
DT	0.444*	0.022	−0.008	−0.048	1	−0.052	0.166	0.094	−0.023	−0.089
Eury	0.248*	0.716*	0.566*	0.717*	−0.052	1	0.455*	0.528*	0.749*	0.497*
Firm	0.056	0.627*	0.555*	0.559*	0.166	0.455*	1	0.412*	0.779*	0.381*
Pro	0.394*	0.51*	0.282*	0.423*	0.094	0.528*	0.412*	1	0.489*	0.125
Spiro	0.075	0.804*	0.725*	0.779*	−0.023	0.749*	0.779*	0.489*	1	0.49*
Ten	0.063	0.347*	0.349*	0.441*	−0.089	0.497*	0.381*	0.125	0.49*	1
(*C*)										
Act	1	0.554*	−0.015	−0.003	0.753*	0.792*	0.485*	0.803*	0.08	−0.266*
Bact	0.554*	1	0.431*	0.386*	0.368*	0.779*	0.908*	0.719*	0.557*	0.139
Chl	−0.015	0.431*	1	0.636*	−0.082	0.337*	0.525*	0.124	0.658*	0.558*
Cren	−0.003	0.386*	0.636*	1	0.033	0.361*	0.422*	0.105	0.539*	0.478*
DT	0.753*	0.368*	−0.082	0.033	1	0.524*	0.298*	0.584*	0.042	−0.233*
Eury	0.792*	0.779*	0.337*	0.361*	0.524*	1	0.699*	0.815*	0.412*	0.067
Firm	0.485*	0.908*	0.525*	0.422*	0.298*	0.699*	1	0.693*	0.587*	0.195
Pro	0.803*	0.719*	0.124	0.105	0.584*	0.815*	0.693*	1	0.183	−0.254*
Spiro	0.08	0.557*	0.658*	0.539*	0.042	0.412*	0.587*	0.183	1	0.664*
Ten	−0.266*	0.139	0.558*	0.478*	−0.233*	0.067	0.195	−0.254*	0.664*	1

Note.—Act, Actinobacteria; Bact, Bacteroidetes; Chl, Chlamydiae; Cren, Crenarchaeota; DT, Deinococcus-Thermus; Eury, Euryarchaeota; Firm, Firmicutes; Pro, Proteobacteria; Spiro, Spirochaetes; Ten, Tenericutes. Asterisk denotes statistical significance (*P* < 0.05, according to the Spearman Correlation test).

Many of the samples analyzed were extracted from a single type of environment, the human gut. In order to examine whether environmental factors impose variability in nucleotide content even within a similar type of environment, we recalculated the above described correlations using only the 111 human gut samples. We found that even when looking only within one type of environment GC-content of different phyla correlates much more frequently than expected by chance (table 2*B*). This was least true for Deinococcus-Thermus that had GC-content levels that correlated significantly with only those of one other phylum, and for Actinobacteria that showed significant correlations with only three other phyla (33%). However, the remaining eight phyla had GC-contents that were significantly correlated to those of other phyla in a minimum of 66.7% of the cases.

Because the human gut samples constituted the majority of our data sets, we removed gut samples from consideration to quantify the contribution of the other environment types to observed correlations (table 2*C*). Once gut samples were removed, the percentage of correlations for all phyla that were significantly correlated ranged between 67% and 89% (table 2*C* and supplementary table S3, Supplementary Material online).

To ensure that the observed correlative effects were not caused by outliers, the analysis was rerun in the same manner as before except that sequences were removed if their GC-content value fell outside the interquartile region for each phylum (see supplementary table S1, Supplementary Material online). Results remained consistent, in that for all phyla, GC-contents significantly correlated with those of other phyla much more frequently than expected by chance (supplementary table S3, Supplementary Material online). For analysis involving all samples, significant correlations were observed in between 55.6% and 100% of the cases, for the different phyla examined (supplementary table S1*A*, Supplementary Material online). When looking at correlations for only human gut datasets, Deinococcus-Thermus (11.1%), Actinobacteria (22.2%), and Tenericutes (22.2%) had the lowest number of significant correlations. For the remaining phyla, significant correlations were observed between 55.6% and 77.8% of cases (supplementary table S1*B*, Supplementary Material online). Excluding the human gut datasets from the analysis, with the exception of Tenericutes (22.2%), the GC-contents of all phyla were significantly correlated between 55.6% and 88.9% of the time (supplementary table S1*C*, Supplementary Material online).

Finally, to examine whether these results could be related to some artifact due to amino acid usage, we annotated the sequences extracted from the different samples and identified protein-coding sequences. This allowed us to calculate the GC-contents of third-codon positions of 4-fold degenerate codons and examine whether these GC-contents were also correlated between phyla across environments. The third-codon positions of 4-fold degenerate codons do not affect the amino acid sequence of a protein. Therefore, their nucleotide content should not be affected by selection at the level of amino acid usage. We found similar results to those reported above. In other words, the GC-content of third-codon positions of 4-fold degenerate codons within protein-coding genes is correlated between phyla across environments much more frequently than the 5% expected by chance (supplementary table S2, Supplementary Material online). When correlations were calculated across all samples, the percentage of significant correlations ranged between 66.7% and 100% for the different phyla examined (supplementary table S2*A*, Supplementary Material online). When only the human gut datasets were considered, the GC-contents of Tenericutes were significantly correlated to those of one phylum (11.1%). The GC-contents of the remaining phyla were found to be significantly correlated to those of between 55.6% and 88.9% of the other phyla (supplementary table S2*B*, Supplementary Material online). Lastly, when excluding the human gut datasets, the GC-contents of 4-fold degenerate 3rd codon positions were significantly correlated for 55.6%–100% of comparisons with the exception of Tenericutes (22.2%) (supplementary tables S2*B* and S3, Supplementary Material online).

Combined, these results demonstrate that whether we look across different environment types, or within one type of environment (the human gut) the GC-contents of different phyla correlate across samples much more frequently than would be expected by chance. These results remain consistent whether the entire sequence is used to calculate GC-content, or whether one uses only the 4-fold degenerate third-codon positions of protein-coding sequences. These results also remain consistent when GC-content outliers are removed from consideration. Such results would only be expected if environmental differences between samples influence the nucleotide content of each phylum in a correlated manner. It is striking that such nucleotide content affecting environmental differences exists not only between diverse environmental types but also within a single type of environment.

### Different Environments Do Not Differ by the Genera They Contain

The correlations in the GC-contents of different phylogenetic taxa across environments were performed at the phyla level. This was due to a constraint imposed on us. Namely, that we cannot reliably classify metagenomic short reads at lower levels of taxonomy. It is thus not feasible for us to examine whether GC-contents of different genera or species correlate across environments. We could therefore not elucidate the taxonomic level at which these correlations are determined. It is for example, possible that a certain environment that favors GC-richness, would not allow certain species to colonize that environment (as we assume that members of a species are too similar to each other to allow for much variation in nucleotide composition). However, genera may be allowed to persist through their members that are more GC-rich. Although we cannot reliably classify individual sequences at taxonomic levels much lower than phylum, we hope that classifications will be reliable enough to allow us to examine which genera are present within each sample (without attempting to estimate their relative abundance). This should allow us to at least examine whether genera tend to be excluded from environments entirely. To do so, we used the relatively unreliable genus-level taxonomic information to examine how similar environments were by the genera they contained. In this respect, we found no large differences between environments. The largest observed difference was between Fish Gut and the Coral, which contained 97.9% of the same genera (supplementary table S4, Supplementary Material online). All remaining samples were at least 98% identical in the identity of the genera they contained. These results imply that selection on nucleotide composition very rarely if at all removes entire genera from the environment. We anticipate that this can be more carefully verified in the near future, when sequencing will yield longer reads which will improve the accuracy of the lower-taxonomic classifications.

### Human Gut Samples that Are Dominated by Actinobacteria Tend to Be More GC-Rich than Other Human Gut Samples

**Fig. 3.—:**
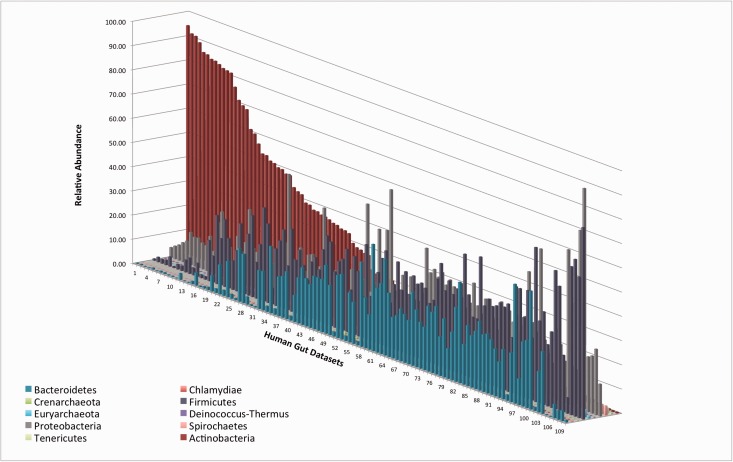
Relative abundance of the ten most abundant phyla in the human gut samples.

When examining the phylogenetic classification of different human gut samples it became clear that these could be divided into two groups: Those that were dominated by Actinobacteria and those in which the most prevalent phyla were Bacteroidetes, Firmicutes, and Proteobacteria (fig. 3). This led us to ask whether the gut samples dominated by Actinobacteria (a highly GC-rich phylum) tended to differ in nucleotide composition from the other samples. To address this question, we ranked the human gut samples separately based on their abundance of Actinobacteria and based on the GC-richness of each of the ten examined phyla. We then found that the 24 (top 22%) samples that had at least 50% relative abundance of Actinobacteria tended to be significantly enriched (when compared with a hypergeometric distribution) for the gut samples with high GC-content in nine out of the ten examined phyla (table 3*A*). We also examined the same question using a different statistical test. We compared the average GC-content of each phylum, within the 22% of gut samples in which Actinobacteria were most abundant, with its average GC-content in the remaining gut samples. This comparison showed that, for seven of the ten examined phyla, GC-content was significantly higher within the Actinobacteria-rich gut samples, compared to the gut samples that were less rich in Actinobacteria (*P* ≪ 0.05, according to a Mann–Whitney test, table 3*B*). Together, these results suggest that environments dominated by Actinobacteria tend to be ones in which selection exists for higher GC-content, across many, if not all phyla.

**Table 3:** Within the Human Gut, High Abundance of the GC-Rich Phylum Actinobacteria Is Associated with Higher GC-Contents of Most Other Phyla: (*A*) Hypergeometric Probability and (*B*) Mann–Whitney–Wilcoxon *P* values for Each Phylum Comparing the Mean GC-Content for the Top 22% Guts with the Bottom 78% Guts Ordered by Actinobacteria Abundance

(*A*)	No. of Top 24 Most Actinobacteria-Rich
	Samples that Are Most GC-Rich (hypergeometric *P* values)
Actinobacteria (GC)	9 (0.026)
Bacteroidetes (GC)	10 (0.008)
Chlamydiae (GC)	11 (0.002)
Crenarchaeota (GC)	11 (0.002)
Deinococcus-Thermus (GC)	9 (0.026)
Euryarchaeota (GC)	16 (0)
Firmicutes (GC)	5 (0.220)
Proteobacteria (GC)	9 (0.026)
Spirochaetes (GC)	11 (0.002)
Tenericutes (GC)	10 (0.008)

**(*B*)**	**Mann–Whitney–Wilcoxon *P* Values**

Actinobacteria	7.4e-6
Bacteroidetes	6.4e-4
Chlamydiae	2.4e-4
Crenarchaeota	1.6e-4
Deinococcus-Thermus	0.257
Euryarchaeota	1.8e-10
Firmicutes	0.198
Proteobacteria	3.0e-4
Spirochaetes	5.2e-4
Tenericutes	0.077

Note.—Number of guts that are dominated by Actinobacteria (24 data sets = ∼22%) of GC-content for each phylum out of the 24 guts with the highest Actinobacteria abundance. In parenthesis is the *P* value of the hypergeometric distribution. This indicates the likelihood that such overabundance is possible by chance.

## Discussion

The results presented here demonstrate that both phylogeny and environment contribute to the determination of prokaryotic nucleotide content.

Our first finding is that different phyla are characterized by different mean GC-contents and that some phyla are characterized by a much broader GC-content range than others. These averages and possible ranges of nucleotide compositions are, to a large extent, maintained across different environments. By examining the sequence variation of fully sequenced members of each phylum, we could show that even phyla with very low levels of nucleotide content variation were often highly variable in their sequences. Thus, low levels of nucleotide content variation within certain phyla could not be explained by low levels of sequence variation within these phyla. A caveat must be added because the range of strains sequenced for each phylum might be limited and because sequences are not selected at random for whole-genome sequencing. It therefore becomes possible that strains exist of those phyla for which we observed low levels of nucleotide content variation that have very different nucleotide contents. However, the range of nucleotide contents observed for each phylum was largely maintained both within fully sequenced genomes and within metagenomic sequences extracted from each of the diverse environments sampled. In order for previously unknown members of a phylum to be outside of the range of GC-contents calculated for that phylum from the metagenomic samples, they would have to be diverged enough from the sequenced members of that phylum so as not to be assigned to the same phyla. This seems less likely to us. At a minimum, we show that for all known members of certain phyla (and for all their relatives that are closely enough related so as to be classified to these phyla from short metagenomic reads), there are low levels of nucleotide content variation that cannot be explained by low levels of sequence diversity.

At the same time, and perhaps even more interestingly, our results show that GC-content varied across environments in a manner that is correlated across prokaryotic phyla. This suggests that whatever force influenced the nucleotide composition of one phylum influenced that of the others. Therefore, variation in nucleotide composition does not stem entirely from differences in phylogeny. Rather, it is probable that the environment exerts some sort of pressure which acts upon all phyla to influence the GC-content in the same direction. We see that environmentally shaped differences in GC-content were apparent when radically different environments were considered and when we examined different samples extracted from a single type of environment (the human gut). Thus, it would appear that the environmental factors which shape nucleotide content vary not only among largely different environments such as soil versus water versus the human microbiome but also within a single type of environment.

It is not yet known what attributes are responsible for determining the range of GC-compositions of the different phyla. It is unlikely that members of a given phylum face consistent selection to maintain similar nucleotide composition ranges across all environments. Therefore, it appears more likely that the average and range of nucleotide compositions adoptable by members of a given phylum would be determined by neutral processes. It has long been assumed that different phyla would have different nucleotide compositions due to differences in mutational biases. However, more recently it was demonstrated that even in GC-rich prokaryotes, mutation is universally AT-biased (Hershberg and Petrov 2010; Hildebrand et al. 2010). Mutation is however, not the only neutral process that could explain differences in prokaryotic nucleotide composition. It is possible that different phyla encode, or in some cases, do not encode the various mechanisms that allow them to modulate nucleotide content in the face of AT-biased mutation. For example, as described in the introduction, it has been demonstrated that gene conversion is GC-biased in many eukaryotes—including humans and other mammals (Duret and Galtier 2009). Additionally, some evidence exists for similar BGC occurring in *Escherichia coli* and additional bacteria (Touchon et al. 2009; Lassalle et al. 2015). Such a BGC mechanism may exist in some but not all prokaryotic phyla, and may be more or less GC-biased in different phyla. Furthermore, different phyla may experience lower or higher recombination rates. Such differences between phyla may lead to differences in their range of possible GC-compositions. However, much more research is necessary to determine why phyla vary so greatly in their nucleotide composition.

This study provides evidence for environmental effects on nucleotide composition. However, we still do not know which environmental factors affect GC-content. Past studies have attempted to link different environmental factors to the nucleotide composition of microbes. One of the most obvious factors thought to influence GC-content was selection on genome stability exerted by high temperatures. Prokaryotes living in high temperatures may need to maintain higher GC-levels, because these may provide better genome stability when temperatures are elevated. Yet, the environmentally influenced differences we observe between nucleotide content within the gut clearly cannot be explained by differences in temperature. After all, different human guts are not expected to vary greatly when it comes to temperature. It also seems unlikely that any other simple environmental factor, such as differences in pH, or salinity would entirely explain the environmentally driven variation in nucleotide content we observed.

Our results demonstrate that selection exerted by the environment likely influences nucleotide composition. This suggests that nucleotide content is a selected trait. The observation that variation in GC-content among different human gut samples is environmentally influenced raises the subject of evolutionary time. Nucleotide content is a relatively slowly evolving trait. Ultimately, a large number of mutations is required to significantly alter GC-composition. If within a certain environment there is selection on prokaryotes to be more GC-rich than in other environments, will prokaryotes have time to evolve toward that GC-content when they are already inside the environment? A second possibility is that selection acts at the moment of introduction into an environment. A specific prokaryote with a nucleotide composition that clashes greatly with the optimal level of a given environment may not be able to colonize that environment in the first place. If this is indeed the case, we would expect to see that certain species of prokaryotes may be excluded from certain environments, due to their mismatched nucleotide composition. It is not currently possible to reliably characterize the phylogeny of short metagenomic reads to the species level. However, when we looked at whether different environments differed greatly in their genera, we found no large differences. We can therefore say that it appears that entire genera are not categorically excluded from environments based on nucleotide composition. However, we cannot currently estimate the extent to which the relative abundance of different genera is influenced by selection at the level of nucleotide composition. Advances in sequencing technology should soon allow for longer read length. This in turn should make it possible to more reliably classify phylogeny within metagenomes down to the genera and even species-level. Once this occurs, we should be able to examine possible fluctuations in the abundance of different genera and how these relate to nucleotide composition. We will also be able to investigate whether certain species are excluded from environments due to their nucleotide composition.

Within human guts, we found significant differences in nucleotide composition between those guts that were dominated by Actinobacteria, and those guts that were dominated by Firmicutes, Bacteroidetes and Proteobacteria. We found that in Actinobacteria-dominated gut samples, other phyla—even those that were AT-rich—tended to be relatively more GC-rich than those in the remaining gut samples. This trend could be explained in two ways. First, it is possible that the high abundance of Actinobacteria itself selects for the GC-richness of other phyla. Second, it is possible that both the higher abundance of Actinobacteria and the elevated GC-content of the other phyla are the result of some characteristic of these guts. For example, it is possible that the environmental factor or factors that select for GC-richness increase the abundance of Actinobacteria (because they are highly GC-rich in general), and skew the remaining, less-abundant phyla to be more GC-rich. If the later scenario is correct, it implies that selection on nucleotide composition may be a factor affecting prokaryotic phylogenetic composition within certain environments.

To conclude, our results demonstrate that although phylogeny is associated with a specific prokaryotic nucleotide composition, the environment strongly influences that composition. Combined, phylogeny and environment direct the GC-content seen in an environment. Different phyla are more or less flexible with regards to the amounts of change in nucleotide composition they can accommodate. Within the range possible for a certain phyla, environment seems to determine whether their GC-content will be higher or lower. Both sharp differences in environment type (e.g., soil vs. aquatic vs. human microbiome) as well as more subtle environment differences (as those observed between different human guts) significantly influence nucleotide content. Thus, the environmental factors affecting nucleotide composition vary not only between highly different environments but also between more similar ones.

## Supplementary Material

Supplementary tables S1–S4 and figures S1–S5 are available at *Genome Biology and Evolution* online (http://www.gbe.oxfordjournals.org/).

Supplementary Data
