# Supplementary material for: Prokaryotic Nucleotide Composition Is Shaped by Both Phylogeny and the Environment
Source: Genome Biol Evol. 2015 Apr 9;7(5):1380–9. doi: 10.1093/gbe/evv063 (PMC4453058; doi:10.1093/gbe/evv063)
Supplement: Supplementary Data [file supp_7_5_1380__index.html]

Prokaryotic nucleotide composition is shaped by both phylogeny and the environment — Prokaryotic Nucleotide Composition Is Shaped by Both Phylogeny and the Environment — Supplementary Data 

# Prokaryotic Nucleotide Composition Is Shaped by Both Phylogeny and the Environment

## Supplementary Data

files

**Files in this Data Supplement:**

- Supplementary Data - pdf file
