# Supplementary material for: Prokaryotic Nucleotide Composition Is Shaped by Both Phylogeny and the Environment
Source: Genome Biol Evol. 2015 Apr 9;7(5):1380–9. doi: 10.1093/gbe/evv063 (PMC4453058; doi:10.1093/gbe/evv063)
Supplement: Supplementary Data [file supp_evv063_Supplementary.pdf]

Supplementary Tables

A	Act	Bact	Chl	Cren	DT	Eury	Firm	Pro	Spiro	Ten
**Act**	1	0.315*	-0.007	0.436*	0.605*	0.58*	0.115	0.517*	-0.292*	-0.026
**Bact**	0.315*	1	0.368*	0.5*	0.308*	0.723*	0.563*	0.576*	0.343*	0.197*
**Chl**	-0.007	0.368*	1	0.218*	0.105	0.273*	0.302*	0.177*	0.392*	0.203*
**Cren**	0.436*	0.5*	0.218*	1	0.276*	0.724*	0.174*	0.331*	-0.044	0.207*
**DT**	0.605*	0.308*	0.105	0.276*	1	0.385*	0.161*	0.333*	-0.067	0.102
**Eury**	0.58*	0.723*	0.273*	0.724*	0.385*	1	0.271*	0.501*	0.055	0.169*
**Firm**	0.115	0.563*	0.302*	0.174*	0.161*	0.271*	1	0.632*	0.622*	0.114
**Pro**	0.517*	0.576*	0.177*	0.331*	0.333*	0.501*	0.632*	1	0.22*	0.057
**Spiro**	-0.292*	0.343*	0.392*	-0.044	-0.067	0.055	0.622*	0.22*	1	0.217*
**Ten**	-0.026	0.197*	0.203*	0.207*	0.102	0.169*	0.114	0.057	0.217*	1
**B**	**Act**	**Bact**	**Chl**	**Cren**	**DT**	**Eury**	**Firm**	**Pro**	**Spiro**	**Ten**
**Act**	1	-0.064	-0.097	0.008	0.3*	0.029	-0.002	0.288*	-0.054	0.032
**Bact**	-0.064	1	0.287*	0.397*	0.093	0.693*	0.397*	0.316*	0.535*	0.178
**Chl**	-0.097	0.287*	1	0.207*	0.013	0.252*	0.236*	-0.01	0.414*	0.136
**Cren**	0.008	0.397*	0.207*	1	0.045	0.443*	0.32*	0.168	0.411*	0.276*
**DT**	0.3*	0.093	0.013	0.045	1	0.026	0.039	-0.158	-0.021	0.08
**Eury**	0.029	0.693*	0.252*	0.443*	0.026	1	0.304*	0.316*	0.587*	0.272*
**Firm**	-0.002	0.397*	0.236*	0.32*	0.039	0.304*	1	0.547*	0.737*	0.079
**Pro**	0.288*	0.316*	-0.01	0.168	-0.158	0.316*	0.547*	1	0.465*	0.008
**Spiro**	-0.054	0.535*	0.414*	0.411*	-0.021	0.587*	0.737*	0.465*	1	0.072
**Ten**	0.032	0.178	0.136	0.276*	0.08	0.272*	0.079	0.008	0.072	1
**C**	**Act**	**Bact**	**Chl**	**Cren**	**DT**	**Eury**	**Firm**	**Pro**	**Spiro**	**Ten**
**Act**	1	0.602*	0.02	0.113	0.707*	0.758*	0.491*	0.727*	0.086	-0.174
**Bact**	0.602*	1	0.33*	0.535*	0.444*	0.898*	0.829*	0.787*	0.518*	0.151
**Chl**	0.02	0.33*	1	0.296*	0.207	0.25*	0.356*	0.273*	0.509*	0.498*
**Cren**	0.113	0.535*	0.296*	1	0.19	0.482*	0.427*	0.343*	0.475*	0.195
**DT**	0.707*	0.444*	0.207	0.19	1	0.544*	0.308*	0.531*	0.173	0.103
**Eury**	0.758*	0.898*	0.25*	0.482*	0.544*	1	0.733*	0.81*	0.368*	0.099
**Firm**	0.491*	0.829*	0.356*	0.427*	0.308*	0.733*	1	0.813*	0.542*	0.068
**Pro**	0.727*	0.787*	0.273*	0.343*	0.531*	0.81*	0.813*	1	0.327*	-0.036
**Spiro**	0.086	0.518*	0.509*	0.475*	0.173	0.368*	0.542*	0.327*	1	0.424*
**Ten**	-0.174	0.151	0.498*	0.195	0.103	0.099	0.068	-0.036	0.424*	1

Table S.1: Spearman correlation coefficients using data filtered to include within-interquartile region values for A) All Environments, B) Human Gut Environment, C) All Environments minus the Human Gut. * Denotes significance. Legend: Act=Actinobacteria, Bact=Bacteroidetes, Chl=Chlamydiae, Cren=Crenarchaeota, DT=Deinococcus-Thermus, Eury=Euryarchaeota, Firm=Firmicutes, Pro=Proteobacteria, Spiro=Spirochaetes, Ten=Tenericutes

**A**	**Act**	**Bact**	**Chl**	**Cren**	**DT**	**Eury**	**Firm**	**Pro**	**Spiro**	**Ten**
**Act**	1	0.49*	0.066	0.081	0.585*	0.583*	0.371*	0.678*	0.247*	-0.041
**Bact**	0.49*	1	0.467*	0.408*	0.504*	0.606*	0.731*	0.697*	0.703*	0.088
**Chl**	0.066	0.467*	1	0.33*	0.102	0.201*	0.475*	0.18*	0.68*	0.236*
**Cren**	0.081	0.408*	0.33*	1	-0.065	0.545*	0.345*	0.243*	0.385*	0.332*
**DT**	0.585*	0.504*	0.102	-0.065	1	0.208*	0.52*	0.524*	0.335*	-0.272*
**Eury**	0.583*	0.606*	0.201*	0.545*	0.208*	1	0.431*	0.608*	0.391*	0.158*
**Firm**	0.371*	0.731*	0.475*	0.345*	0.52*	0.431*	1	0.565*	0.724*	0.152*
**Pro**	0.678*	0.697*	0.18*	0.243*	0.524*	0.608*	0.565*	1	0.437*	-0.018
**Spiro**	0.247*	0.703*	0.68*	0.385*	0.335*	0.391*	0.724*	0.437*	1	0.207*
**Ten**	-0.041	0.088	0.236*	0.332*	-0.272*	0.158*	0.152*	-0.018	0.207*	1
**B**	**Act**	**Bact**	**Chl**	**Cren**	**DT**	**Eury**	**Firm**	**Pro**	**Spiro**	**Ten**
**Act**	1	0.237*	0.168	0.128	0.339*	0.361*	0.092	0.402*	0.221*	0.204*
**Bact**	0.237*	1	0.504*	0.637*	0.307*	0.544*	0.513*	0.513*	0.72*	-0.094
**Chl**	0.168	0.504*	1	0.421*	0.042	0.392*	0.408*	0.042	0.624*	-0.072
**Cren**	0.128	0.637*	0.421*	1	0.063	0.581*	0.501*	0.318*	0.688*	0.084
**DT**	0.339*	0.307*	0.042	0.063	1	-0.007	0.327*	0.219*	0.188*	-0.088
**Eury**	0.361*	0.544*	0.392*	0.581*	-0.007	1	0.356*	0.425*	0.681*	0.052
**Firm**	0.092	0.513*	0.408*	0.501*	0.327*	0.356*	1	0.293*	0.697*	0.063
**Pro**	0.402*	0.513*	0.042	0.318*	0.219*	0.425*	0.293*	1	0.462*	0.134
**Spiro**	0.221*	0.72*	0.624*	0.688*	0.188*	0.681*	0.697*	0.462*	1	0.032
**Ten**	0.204*	-0.094	-0.072	0.084	-0.088	0.052	0.063	0.134	0.032	1
**C**	**Act**	**Bact**	**Chl**	**Cren**	**DT**	**Eury**	**Firm**	**Pro**	**Spiro**	**Ten**
**Act**	1	0.684*	-0.032	-0.023	0.894*	0.837*	0.64*	0.885*	0.416*	-0.15
**Bact**	0.684*	1	0.325*	0.313*	0.65*	0.815*	0.91*	0.785*	0.726*	0.16
**Chl**	-0.032	0.325*	1	0.489*	0.023	0.185	0.435*	0.144	0.613*	0.251*
**Cren**	-0.023	0.313*	0.489*	1	0.087	0.326*	0.33*	0.138	0.39*	0.208
**DT**	0.894*	0.65*	0.023	0.087	1	0.752*	0.651*	0.848*	0.386*	-0.224
**Eury**	0.837*	0.815*	0.185	0.326*	0.752*	1	0.756*	0.845*	0.598*	0.02
**Firm**	0.64*	0.91*	0.435*	0.33*	0.651*	0.756*	1	0.78*	0.785*	0.208
**Pro**	0.885*	0.785*	0.144	0.138	0.848*	0.845*	0.78*	1	0.547*	-0.011
**Spiro**	0.416*	0.726*	0.613*	0.39*	0.386*	0.598*	0.785*	0.547*	1	0.286*
**Ten**	-0.15	0.16	0.251*	0.208	-0.224	0.02	0.208	-0.011	0.286*	1

Table S.2: Spearman correlation coefficients using 3rd codon position data of 4-fold redundant amino acids of for A) All Environments, B) Human Gut Environment, C) All Environments minus the Human Gut. * Denotes significance. Legend: Act=Actinobacteria, Bact=Bacteroidetes, Chl=Chlamydiae, Cren=Crenarchaeota, DT=Deinococcus-Thermus, Eury=Euryarchaeota, Firm=Firmicutes, Pro=Proteobacteria, Spiro=Spirochaetes, Ten=Tenericutes

Groups	All			Human Gut			Non-Human Gut		
**Conditions**	**O**	**IQR**	**T**	**O**	**IQR**	**T**	**O**	**IQR**	**T**
**Act**	6	5	6	6	2	3	6	6	7
**Bact**	8	8	8	8	6	7	8	9	9
**Chl**	5	7	6	5	5	7	7	7	7
**Cren**	5	6	6	6	6	7	7	8	8
**DT**	6	5	6	5	1	1	7	6	5
**Eury**	7	8	8	7	7	8	9	8	8
**Firm**	8	8	8	7	6	7	9	7	7
**Pro**	6	8	6	7	5	7	8	8	8
**Spiro**	9	7	6	8	6	7	9	6	8
**Ten**	2	2	6	1	2	6	6	5	7
**Total**	68	69	72	65	47	61	83	76	79

Table S.3: Number of significant Spearman correlations coefficients for All Environments, Human Gut Environment, and All Environments minus the Human Gut. For conditions, O=original data, IQR=Interquartile Region (all GC values outside of IQR were excluded), and T=3rd Codon Position. Legend: Act=Actinobacteria, Bact=Bacteroidetes, Chl=Chlamydiae, Cren=Crenarchaeota, DT=Deinococcus-Thermus, Eury=Euryarchaeota, Firm=Firmicutes, Pro=Proteobacteria, Spiro=Spirochaetes, Ten=Tenericutes

	**C. Cecum**	**C. Soil**	**Coral**	**Cow Rumen**	**Dental Plaque**	**Fish Slime**	**Gut Fish**	**Gut Human**	**Micro**	**Tundra**	**Water Marine**	**Water Mine**	**Water Pond Fresh**	**Water Pond Saline**
**C. Cecum**	1	1	0.989	1	1	0.996	0.983	1	1	1	1	1	1	1
**C. Soil**	1	1	0.989	1	1	0.996	0.983	1	1	1	1	1	1	1
**Coral**	0.989	0.989	1	0.989	0.989	0.989	0.979	0.989	0.989	0.989	0.989	0.989	0.989	0.989
**C. Rumen**	1	1	0.989	1	1	0.996	0.983	1	1	1	1	1	1	1
**D. Plaque**	1	1	0.989	1	1	0.996	0.983	1	1	1	1	1	1	1
**Fish Slime**	0.996	0.996	0.989	0.996	0.996	1	0.987	0.996	0.996	0.996	0.996	0.996	0.996	0.996
**Gut Fish**	0.983	0.983	0.979	0.983	0.983	0.987	1	0.983	0.983	0.983	0.983	0.983	0.983	0.983
**G. Human**	1	1	0.989	1	1	0.996	0.983	1	1	1	1	1	1	1
**Micro**	1	1	0.989	1	1	0.996	0.983	1	1	1	1	1	1	1
**Tundra**	1	1	0.989	1	1	0.996	0.983	1	1	1	1	1	1	1
**W. Marine**	1	1	0.989	1	1	0.996	0.983	1	1	1	1	1	1	1
**Water Mine**	1	1	0.989	1	1	0.996	0.983	1	1	1	1	1	1	1
**Pond Fresh**	1	1	0.989	1	1	0.996	0.983	1	1	1	1	1	1	1
**Pond Saline**	1	1	0.989	1	1	0.996	0.983	1	1	1	1	1	1	1

Table S.4: The pair-wise Jaccard similarity coefficient values indicating the similarity between two environments based on the types of prokaryotes (genus-level) contained within an environment. The lowest level of similarity is 97.9%. This is a single occurrence and is present between fish gut and coral environments. All other values are at or above 98%. C. Cecum = Chicken Cecum, C. Soil = Contaminated Soil, C. Rumen = Cow Rumen, D. Plaque = Dental Plaque, G. Human = Gut Human, Micro=Microbialites, W. Marine = Water Marine.

Supplementary Figures

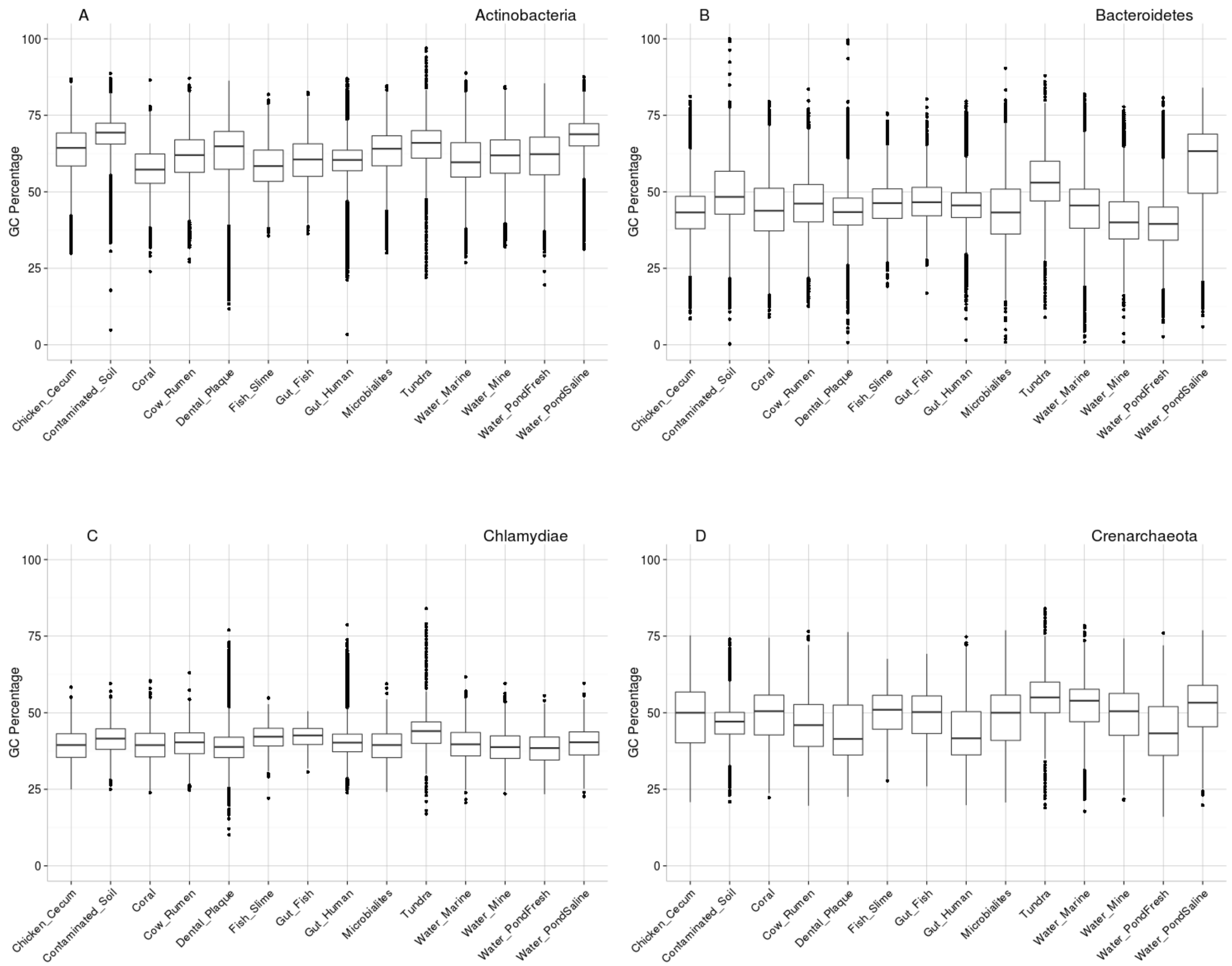

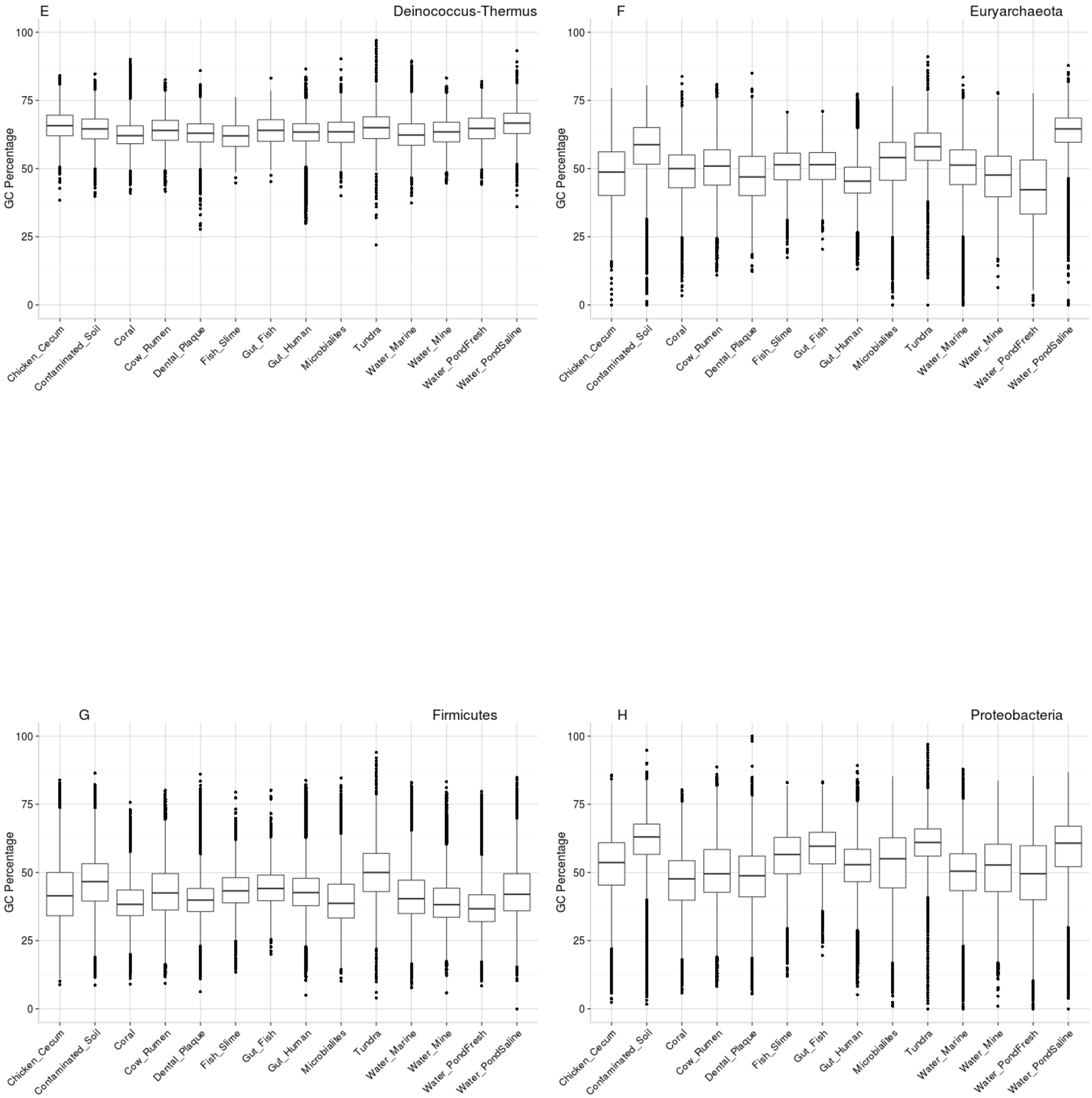
E
Deinococcus-Thermus
F
Euryarchaeota
G
Firmicutes
H
Proteobacteria
GC Percentage
100
75
50
25
0
Chicken_Cecum
Contaminated_Soil
Coral
Cow_Rumen
Dental_Plaque
Fish_Slime
Gut_Fish
Gut_Human
Microbialites
Tundra
Water_Marine
Water_Mine
Water_PondFresh
Water_PondSaline

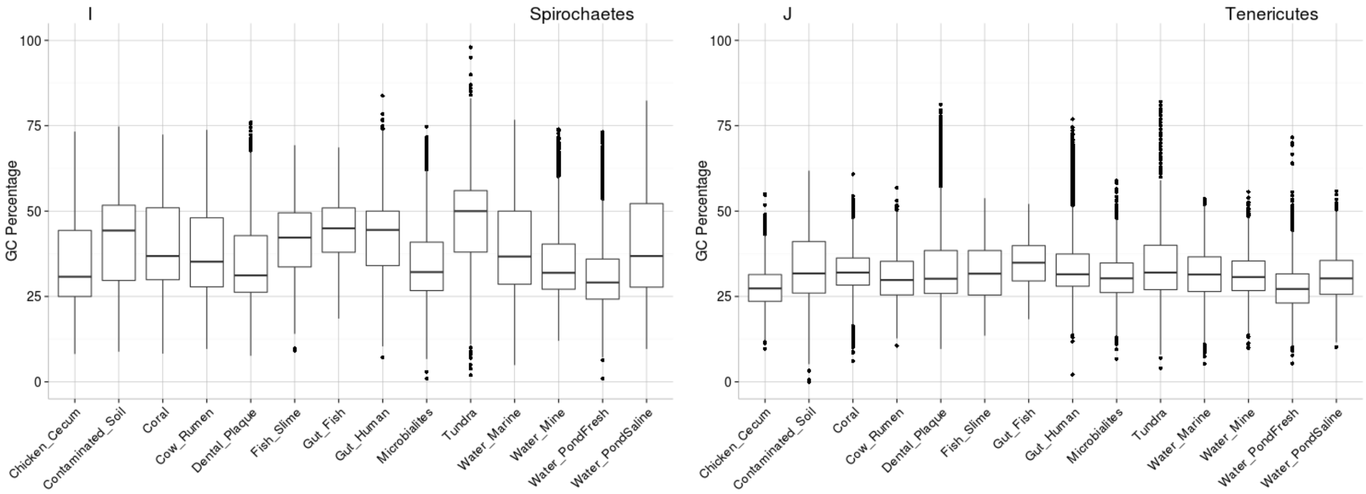

Figure S.1: GC-levels for each phylum across all environments. A=Actinobacteria, B=Bacteroidetes, C=Chlamydiae, D=Crenarchaeota, E=Deinococcus-Thermus, F=Euryarchaeota, G=Firmicutes, H=Proteobacteria, I=Spirochaetes, J=Tenericutes

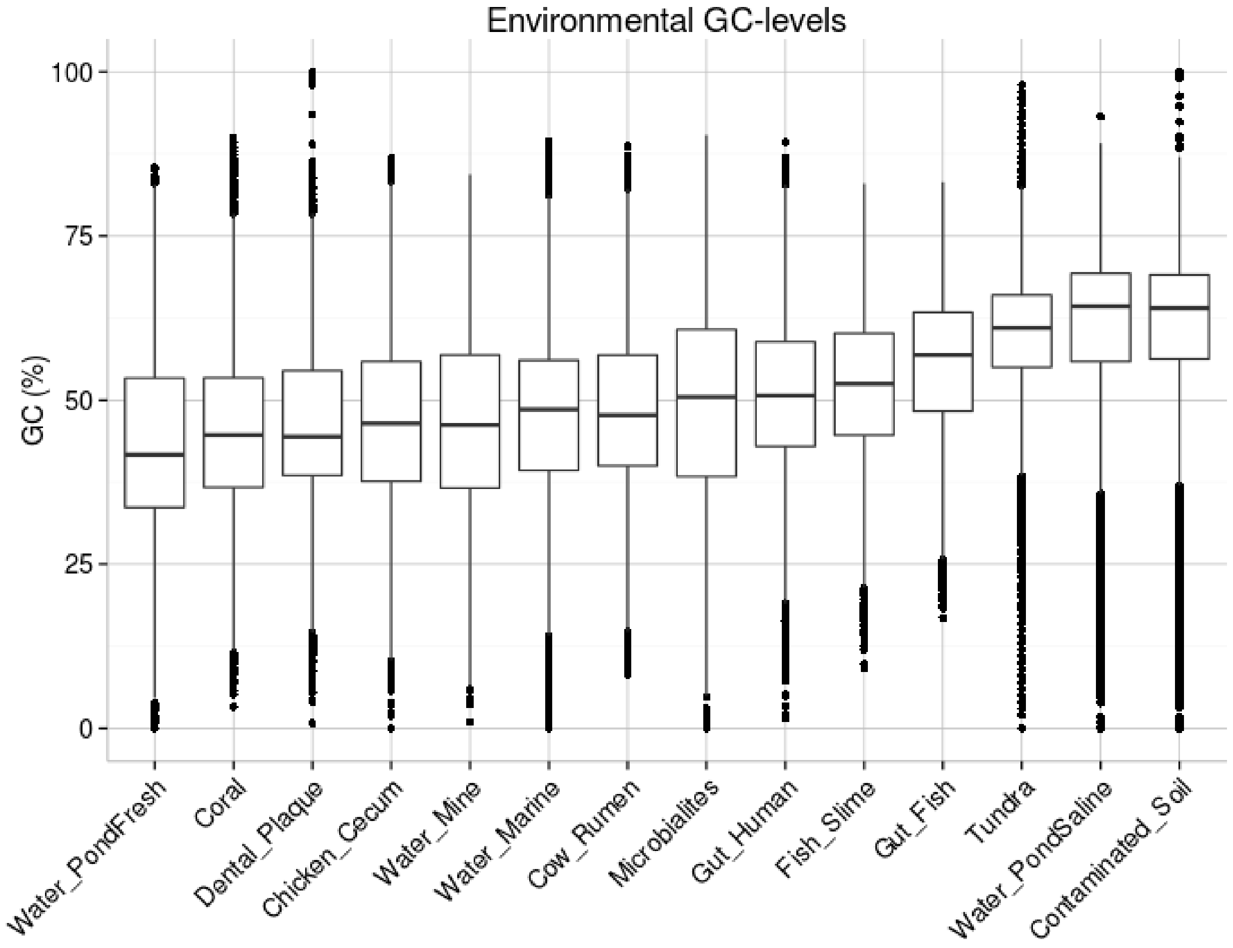

Figure S.2: Boxplot of sequence GC-levels by environment.

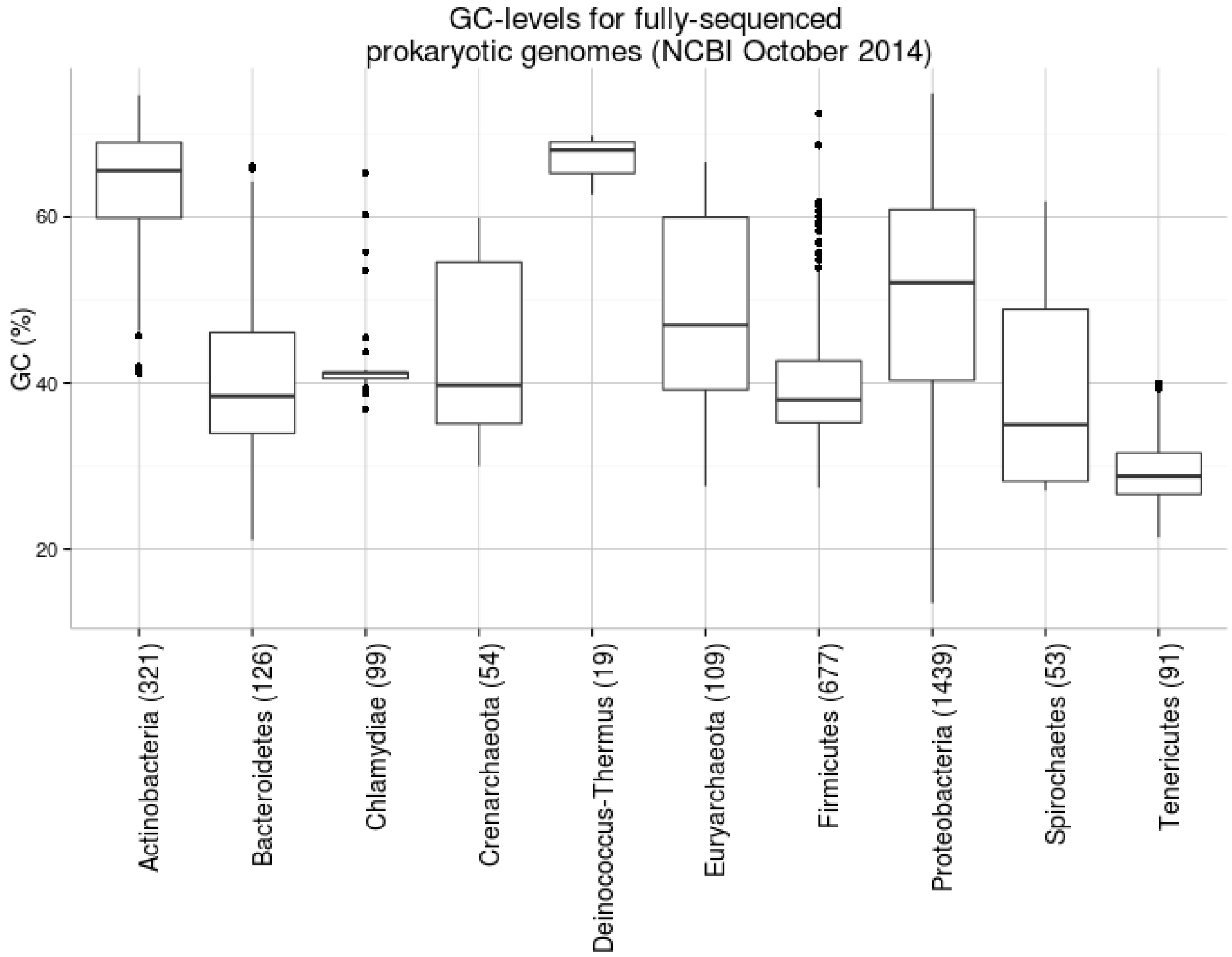

Figure S.3: GC vs. Phylum. GC-levels for fully-sequenced prokaryotic genomes from NCBI as of October 2014.

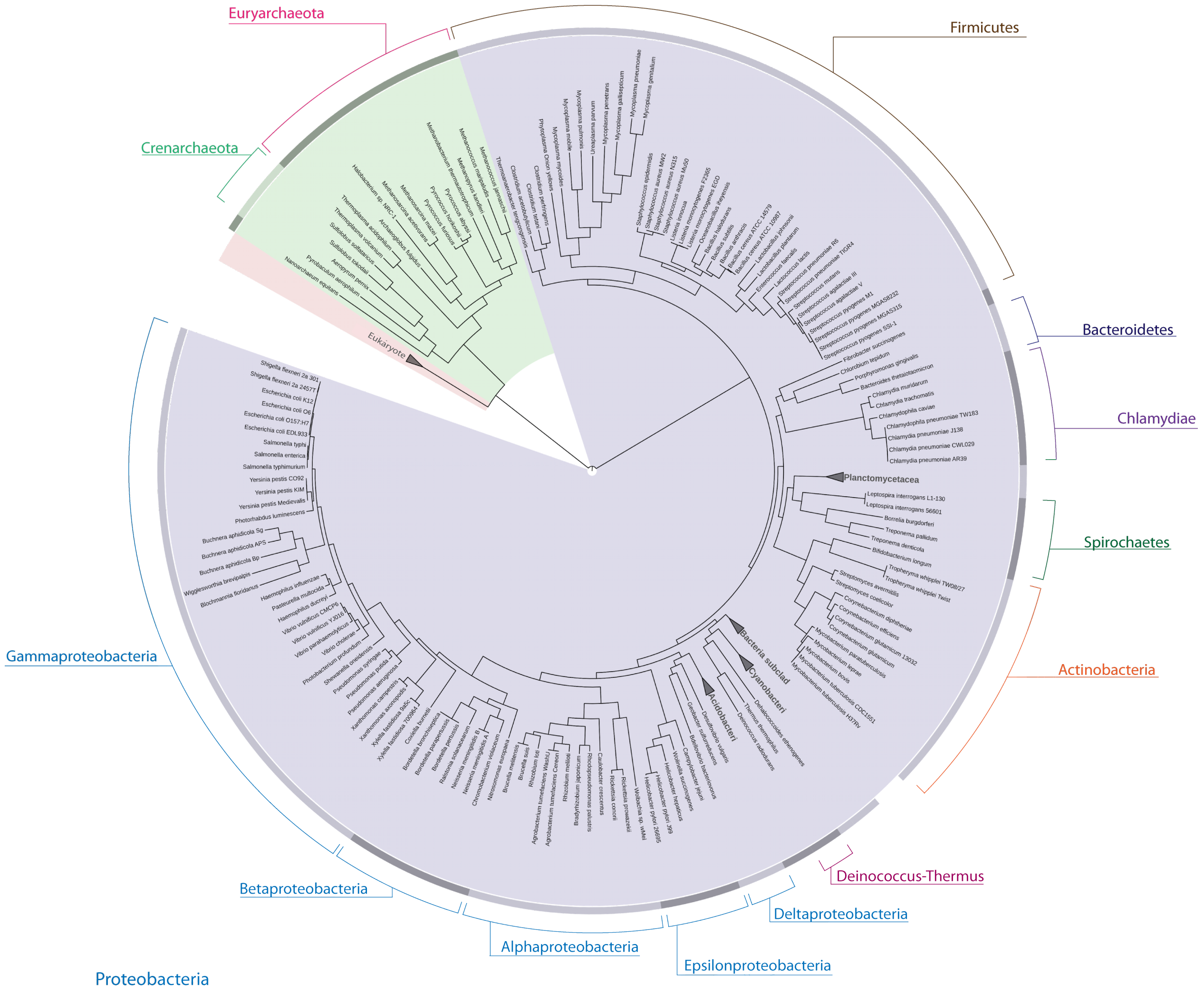

Figure S.4: Phylogenetic tree comprised of species from the phyla used in our analysis. Color (inner circle) legend: Pink = Eukaryotes, Green = Archaea, Light Violet = Bacteria.

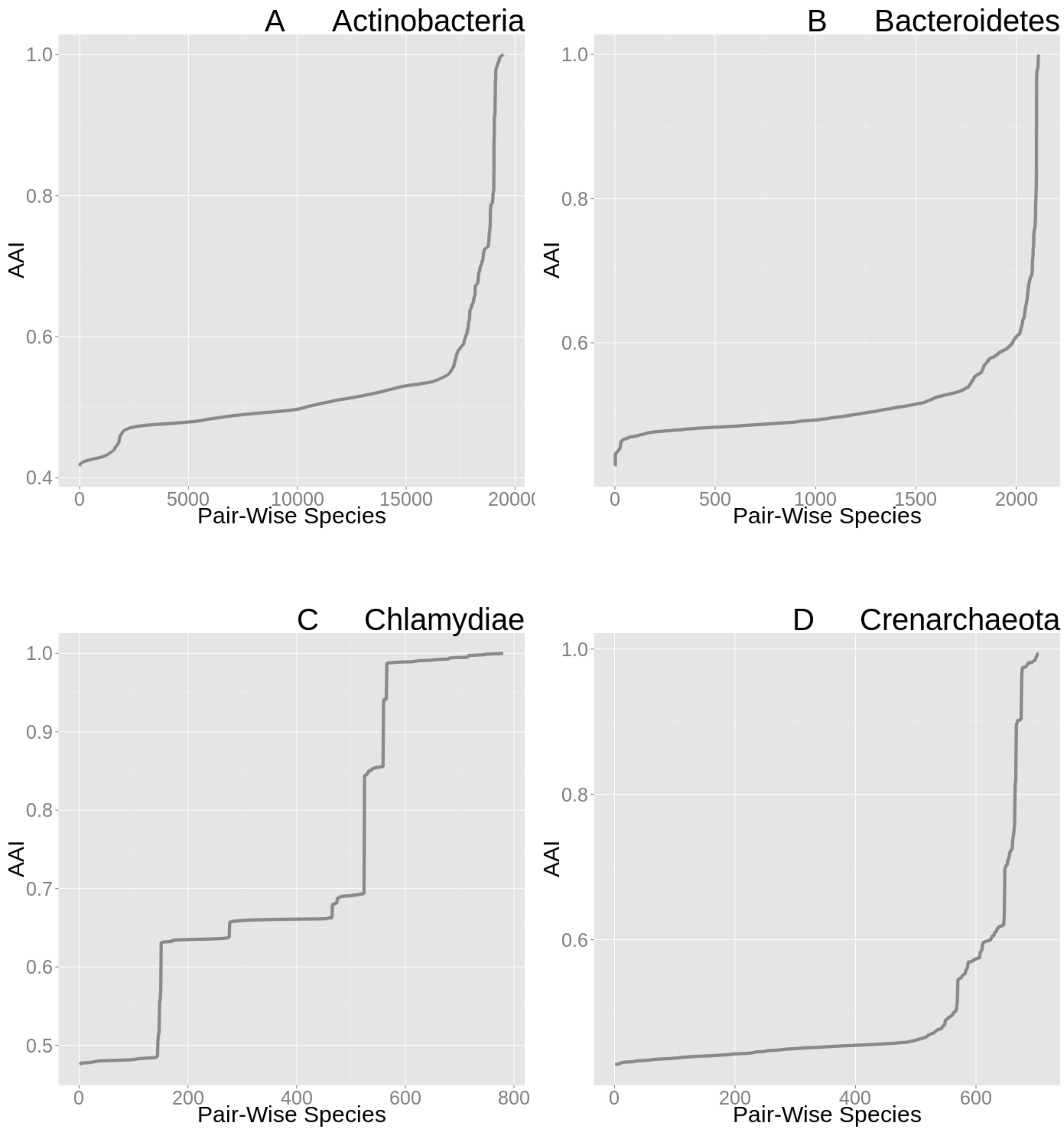
A Actinobacteria
AAI
Pair-Wise Species
B Bacteroidetes
AAI
Pair-Wise Species
C Chlamydiae
AAI
Pair-Wise Species
D Crenarchaeota
AAI
Pair-Wise Species

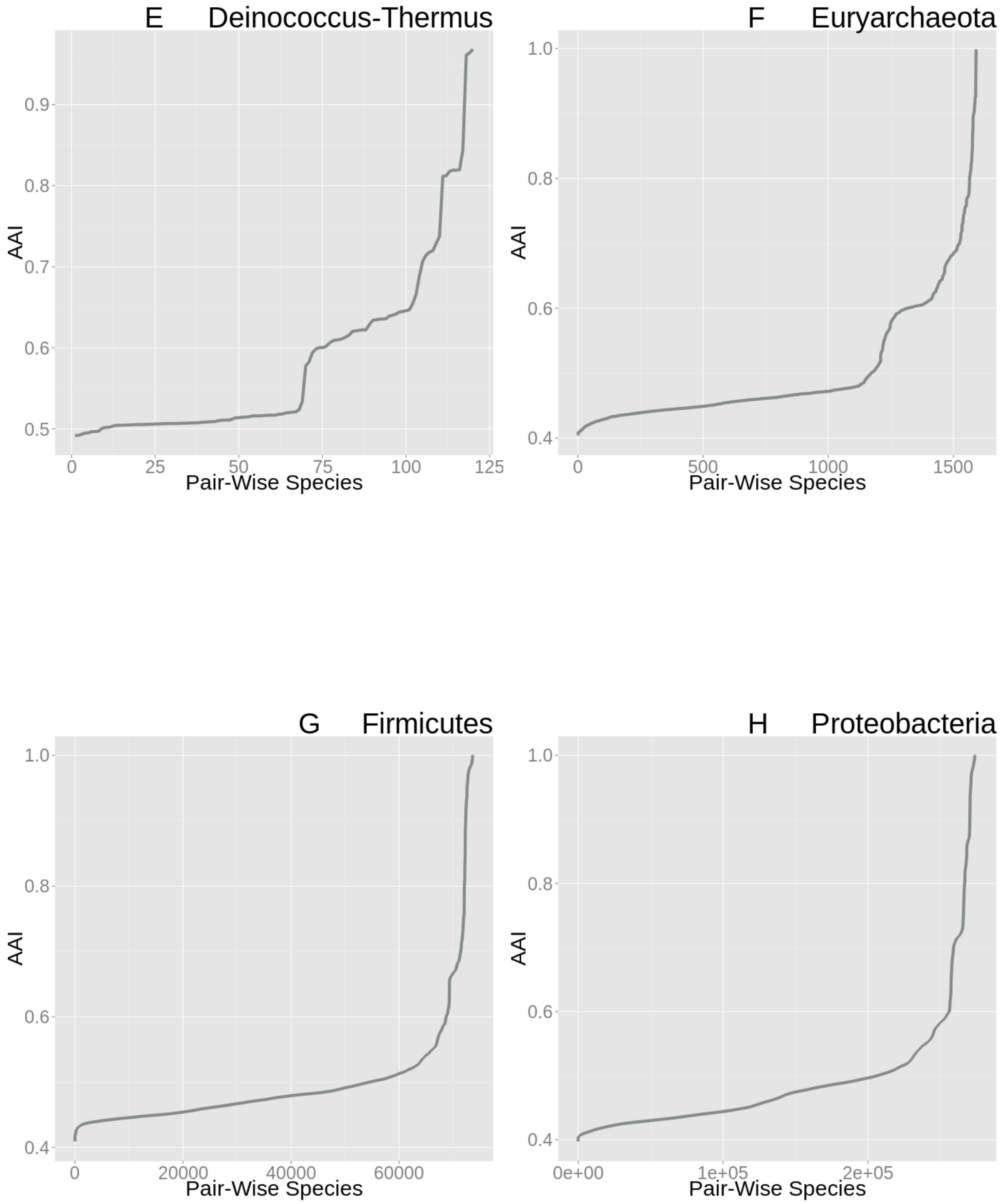
E Deinococcus-Thermus
AAI
0.5
0.6
0.7
0.8
0.9
0
25
50
75
100
125
Pair-Wise Species
F Euryarchaeota
AAI
0.4
0.6
0.8
1.0
0
500
1000
1500
Pair-Wise Species
G Firmicutes
AAI
0.4
0.6
0.8
1.0
0
20000
40000
60000
Pair-Wise Species
H Proteobacteria
AAI
0.4
0.6
0.8
1.0
0e+00
1e+05
2e+05
Pair-Wise Species

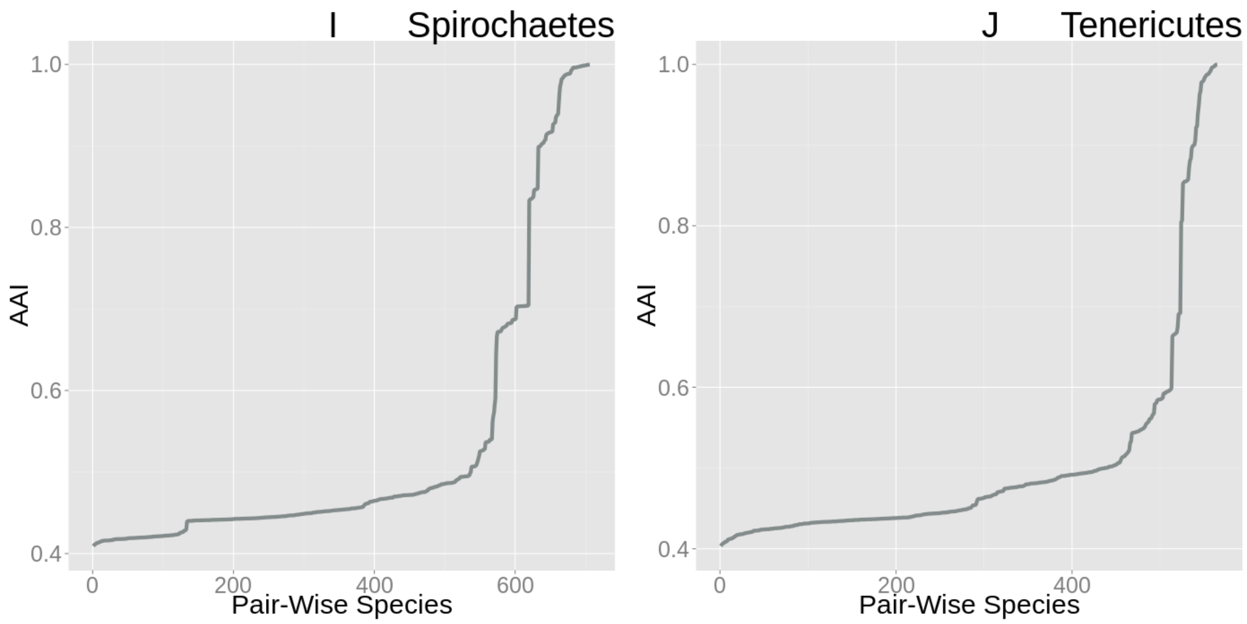

Figure S.5: AAI values calculated for all genomes sharing greater than 80% 16S identity. A=Actinobacteria, B=Bacteroidetes, C=Chlamydiae, D=Crenarchaeota, E=Deinococcus-Thermus, F=Euryarchaeota, G=Firmicutes, H=Proteobacteria, I=Spirochaetes, J=Tenericutes
